# Well-being in the workplace through interaction between individual characteristics and organizational context

**DOI:** 10.3402/qhw.v8i0.19823

**Published:** 2013-02-18

**Authors:** Gianluca Biggio, Claudio G. Cortese

**Affiliations:** 1 Department of Communication Sciences, University of Tuscia, Viterbo, Italy; 2 Department of Psychology, University of Turin, Turin, Italy

**Keywords:** Communication, emotion in organizations, employee attitudes, organizational behaviour, organizational psychology, organizational well-being, personality, work environment

## Abstract

Well-being in the workplace is considered by many authors to be the outcome of the interaction between individual characteristics and those of the working and organizational environment. This study aims to understand the significance attributed to the concept of well-being in the workplace by employees, its influencing factors, and, among those, the role of individual psychological characteristics. The research was conducted on a sample of 72 employees using a qualitative approach based on focus groups and individual interviews. Data analysis was performed by a paper and pencil technique. The focus groups and interviews collected 628 statements, which were divided into three main areas: meaning of well-being in the workplace (248), any kind factors that affect well-being in the workplace (158), and individual characteristics that affect well-being in the workplace (222). The individual characteristics identified by the participants as capable of influencing well-being in the workplace include being positive, communication, management of difficulties and conflicts, socio-emotional skills, and values. The research was limited by the participants involved and by the sole use of the paper and pencil technique of data analysis. Results highlight that well-being in the workplace does not depend exclusively on external conditions in terms of the working and organizational environment within which the individual operates: so, it could be promoted not only from above, through actions by management, but also from below, influencing individual traits and behaviours. Results would be useful for developing training, workplace counselling, and organizational development activities aimed to support small groups, leaders, and other strategic players in the construction of the subsystems of well-being in the workplace.

The theme of well-being in the working environment can be observed from a particular point of view, stemming from which well-being itself is recognizable as the result of interaction between the characteristics of the individual and those of the working context. In other words, contrary to the assumption according to which well-being in the workplace depends exclusively on external conditions in terms of the working and organizational environment within which the individual operates (Burke, 1993; Guest, 2002; Lawson, Noblet, & Rodwell, 2009), the point of view referred to suggests that individual characteristics can play an active part in the development of well-being.

Exploring the views of the authors who have dealt with well-being as a result of the interaction between subjective factors and characteristics of the workplace, it is possible to recognize a common root in interactional theories, which considers a person–organization fit as being crucial in generating well-being (Alvesson & Willmott, 2002; Cable & Judge, 1996; Mininni, Manuti, Scardigno, & Rubino, 2010), and three main aspects of study in which this general approach has declined: the study of job satisfaction, positive emotions, and relational interaction.

## Job satisfaction

An initial approach, which has permitted the analysis of the relationship between well-being in the workplace and subjectivity, was the study of job satisfaction (Harris, Daniels, & Briner, 2003; Judge, Heller, & Klinger, 2008; Ter Doest, Maes, Gebhardt, & Koelewijn, 2006). According to Harter, Schmidt, and Keyes (2003), two lines of research characterize this approach. The first is connected to the theory of the person–environment fit (French, Caplan, & Van Harrison, 1982), in which well-being is connected to the presence of appropriate requests to the individual by the organization. A second line of research—the closest to our hypothesis—relates the performance and the quality of life of people with the presence of positive emotional states and satisfying relationships within the work environment (Isen, 1987; Warr, 1999). When their environment encourages people to seek out challenging or significant tasks, according to Csikszentmihályi (1997), optimal conditions exist for mutual well-being between individuals and the work environment. The assumption in this method of framing the problem is that well-being in the workplace is related to job satisfaction, and this, in turn, is stimulated by the subjective ability to find a positive personal equilibrium within organizational interaction.

Brunstein, Schultheiss, and Grässmann (1998) emphasize the importance of the willingness to define suitable personal objectives (goals) with the scope of encouraging personal well-being. However, the subjective capacity to establish a satisfactory psychological contract with the organizational environment seems to be linked with well-being in the workplace (Guest & Conway, 2002); according to these authors, in fact, the psychological contract that people are able to subjectively maintain has become a formula widely used in research and has proven useful to explain many employees’ behaviours, including attitudes towards health and well-being.

## Positive emotions

A second way of viewing the relationship between individual characteristics and well-being in work settings has as its cornerstone, the hypothesis that positive emotions generate well-being (De Neve & Copper, 1998; Fineman, 2006; Hochwarter & Thompson, 2010; Linley, Harrington, & Garcea, 2010). Assuming that interaction between a person's subjective aspects and the organization can have positive outcomes (O'Brien-Wood, 2001), we can make reference to ample documentation that examines the issue of self-confidence as a resource both for the well-being of the individual and the organization (Pierce & Gardner, 2004; Zapf, 2002).

Di Nuovo and Zanchi (2008) confirm that employee participation in the company's mission, positive emotions, emotional climate, and the sense of belonging within the organization are interdependent. Other authors (Feldt, Mäkikangas, & Aunola, 2006; Kalimo, Pahkin, & Mutanen, 2002; Pulkkinen, Feldt, & Kokko, 2006), referring to the theory of control of the emotions (Gross, 1998, 2006), highlight how emotional control based on a sense of coherence, optimism, and self-esteem plays a developmental role with respect to a series of social interactions, including work. Their longitudinal studies on emotive control have found that positive emotions in adolescence have a beneficial effect on scholastic and subsequent workplace integration. Custers and Aarts (2005) argue that positive affection plays a key role as a motivator in the unconscious disposition towards the pursuance of objectives, thereby contributing to a better relationship with the working environment, as reported on the relationship between job satisfaction and well-being by Wright, Cropanzano, and Bonett (2007).

An additional contribution to the hypothesis of positive emotions as generators of well-being in the workplace comes from the cultural analysis of Alvesson and Willmott (2002): their study underlines how a sense of internal coherence and a positive self-regard are factors which facilitate a positive process of organizational control, resulting in an improvement in the climate of the working environment. Another perspective on positive emotions is also underlined by the American school of counselling. Beginning with the social learning theory (Bandura, 1986), counselling has developed a vision of social and cognitive satisfaction within scholastic contexts and later in the employment context. These authors provide a theoretical perspective that shows the integration between cognitive, social, and personality variables, the latter related to the tendency to express positive emotions, as being effective in promoting well-being within specific areas of life such as work and school (Lent, 2004, 2008; Lent & Brown, 2006; Sheu & Lent, 2009). Furthermore, Lent and Fouad (2011) also support a correlation between positive emotions which are present in the self and an appropriate cognitive and social development of the individual.

## Relational interaction

A third approach that theorizes the possibility of an individual to generate well-being in the workplace is connected with the study of interpersonal skills (Bambacas & Patrickson, 2008; Fligstein, 1997; Purkiss, Rossi, Glendon, Thompson, & Myors, 2008) and especially with the attitude towards extroversion and active relational interaction. Kamdar and Van Dyne (2007) have observed that social exchange supported by sociability produces effects of organizational citizenship, improving employees’ performance of tasks. Ryan and Deci (2000) have verified that the innate psychological needs for competence, autonomy, and openness in relationships, if met, will provide greater self-motivation and, if obstructed, can lead to a decrease in motivation and well-being. Butler and Waldroop (2004) identified four relational dimensions within the work (influence, interpersonal facilitation, relational creativity, leadership, and team) highly correlated in creating satisfaction, performance, and organizational ability at work.

Hughes (2005) reported a study that showed that extroverts experienced less fatigue and stress at work. Some authors emphasize the relationship between the Big Five traits (including extroversion, agreeableness, openness) and psychological well-being (Grant, Langan-Fox, & Anglim, 2009; Haslam, Whelan, & Bastian, 2009). As noted previously, individual psychological well-being may contribute to the welfare of the organization by improving job performance and group atmosphere. Kumar, Bakhshi, and Rani (2009) explore—using the Big Five—the link between personality and organizational citizenship behaviours (OCBs), finding that extroversion and agreeableness in interpersonal relationships support OCBs.

George and Jones (1997) sustain that extra role behaviour, such as spontaneity in interpersonal relationships, helps to create an isomorphic relationship of spontaneity and well-being, including at the organizational level. According to Ferris, Perrewé, Anthony, and Gilmore (2000) and Perrewé, Ferris, Funk, and Anthony (2000) extroversion, openness, respect, confidence, trust, and sincerity are political skills that will improve relations within the team by reducing stress in the workplace and are predictive of ability for success in a wide range of jobs in highly dynamic organizational environments that require flexibility.

Openness to emotional expression and the capacity to create a playful group identity are connected to well-being in temporary groups (Terrion & Ashforth, 2002), while the ability to have open relationships and express one's personal characteristics is seen as a factor in subjective well-being and at the same time as a factor capable of increasing the productivity of those who work in social service organizations (Graham & Shier, 2010, 2011).

## Objectives

This research has proposed three different objectives: (a) explore the meaning attributed to the concept of well-being in the workplace by a group of employees, (b) identify which factors of any kind are perceived as capable of influencing well-being in the workplace, and (c) verify whether individual characteristics are perceived as capable of influencing well-being in the workplace and which appear to have greater power to do so.

## Method

### Procedure

The research, given its descriptive purposes, was carried out from a qualitative perspective and was based both on the use of focus groups (Krueger & Casey, 2000) and semi-structured individual interviews (Gabriel, 2000; Murray, 2002).

Focus groups, lasting 3 h each, were conducted by a pair formed of an interviewer and an observer, using a set of three open-ended questions that explored the perception of the group in relation to three aspects: (a) the meaning of well-being in the workplace (“What does ‘well-being in the in the workplace’ mean to you? How could you describe it?”), (b) the factors that affect well-being in the workplace (“What are the any kind factors that you believe can influence the creation of well-being in the workplace?”), and (c) the presence of (other) individual characteristics, besides those mentioned above, which can affect well-being in the workplace (“Do you particularly believe individual characteristics exist that may influence the creation of well-being in the workplace? If you agree, which are they?”).

As can be seen, the second question left the participants free to indicate, among the factors that influence well-being in the workplace, both organizational and individual characteristics, while the third question led subjects to specifically consider the characteristics of the individual type. In the event that this had already been mentioned in the answers to the second question, the participants were asked to be more specific and possibly mention others. If on the other hand such characteristics had not been mentioned, they were asked if they were deemed capable of influencing well-being, and—if affirmative—to specify and describe.

In total, seven focus groups were carried out, which took place in a meeting room, protected from external interference, within two organizations.

Individual interviews, lasting an hour and half, were conducted by a pair composed of an interviewer and an observer using the same questions of the focus groups. In addition, during the interviews, the interviewer asked the participants to provide examples of stories related to events of particular relevance to their well-being, in order to clarify their statements. In total, nine individual interviews were carried out, which took place within a third organization in a quiet meeting room far from company operations.

The focus groups and interviews were recorded and transcribed in full, except for information that could lead to the recognition of the participants. Participants gave their informed consent to participation in the research and were assured anonymity in the data which emerged. In addition, focus groups and interviews were made with the consent of the Human Resources Directors of the three organizations in which research was conducted and also of the participants’ managers.

### Participants

Sixty-three participants took part in the focus group: 36 employees of the National Health Service (four focus groups) and 27 employees of a private company (three focus groups). Nine individual interviews were carried out with nine employees of a multinational company. The companies were chosen randomly within a set of organizations, located in central Italy, which had expressed their interest, so as to favour the plurality of working contexts investigated. Managerial roles were excluded and the subjects were composed of professional employees (e.g., doctors, nurses, and human resources employees) and team leaders (e.g., coordinators, personnel administrators, and corporate project leaders). The socio-demographic characteristics of the 72 subjects who took part in the research are shown in Table I.

**Table I:** Socio-demographic characteristics of the participants.

Characteristic	*n*	%
Age		
Up to 34	12	16.7
35–39	17	23.6
40–44	19	26.4
45–49	16	22.2
50 and above	8	11.1
Gender		
Female	37	51.4
Male	35	48.6
Role		
Team leader	24	33.3
Professional employee	48	66.7

### Data analysis

The content analysis of material collected through focus groups and interviews was conducted using a paper and pencil technique. This analysis took place in three distinct phases: (a) in the first phase, the perceptions of the meaning of well-being in the workplace were searched out; (b) in the second phase, the general perceptions of factors influencing well-being were searched out; (c) in the third phase, the perceptions of individual characteristics influencing well-being were searched out. (Kyngas & Vanhanen, 1999).

Initially, a database of statements relating to each of the three aspects was created. Each statement was encoded by identifying the central element in the classification (e.g., “reduction of hierarchical barriers”). The encoding was made independently by each of the three researchers who participated in the study. In this way, each researcher had read all of the material gathered through research. The encodings of the three researchers were compared in order to reach a final result. In the event of disagreement, the case in question was discussed, in order to achieve a convergence of views.

Various elements emerged (14 for the perception of the meaning of well-being in the workplace, 9 for the perception of factors influencing well-being in the working context and 14 for the perception of individual characteristics that influence well-being in work contexts), which were subsequently moved to the more general categories to which they belonged (e.g., “participatory hierarchy”). This step was carried out independently by each of the three researchers, who then compared their categorizations to reach a final agreed choice. Two weeks after the conclusion of the data analysis, transcriptions from two focus groups and an interview were once more encoded, again independently by the three researchers, with a categorical confirmation of stability.

Finally, the analysis of illustrative stories told by the subjects during the interviews made it possible to make some considerations about the dynamics of the concept of well-being in the workplace.

## Results

The focus groups and interviews collected 628 statements, which were divided into three areas: (a) the significance of well-being in the workplace (248 statements), (b) every kind of factors that affect well-being in the workplace (158 statements), and (c) individual characteristics that affect well-being in the workplace (222 statements).

As shown in Tables II, III and IV, the perceptions expressed through a set of categories have been listed in each area. For each category, moreover, it was possible to further differentiate specific elements that provide a more detailed description of the perceptions of individuals.

**Table II:** The meaning of well-being in the workplace.

Category	*n*	Specific elements (*n*)
		Transparency in communication between colleagues (26)
		Staying within the boundaries (20)
Acceptance of the rules	76	Clarity in the definition of rights and duties by the company (16)
		Avoidance of disputes in business relationships (14)
		Sharing the company vision (22)
		Participatory leadership (18)
Participatory hierarchy	67	Reduction of hierarchical barriers (15)
		Leading by example (12)
		Knowing how to listen (23)
Positive relations and working climate	58	Being able to rely on the group (19)
		Mental flexibility (16)
Appreciation of the value of work	47	Being motivated by work content (20)
		Economic reward (16)
		Job rotation and change in routine role (11)

**Table III:** Factors which influence organizational well-being.

Category	*n*	Specific elements (*n*)
		Respect (24)
Values	59	Humility (19)
		Transparency and exchange of information (16)
		Fluid organization (22)
Organizational functioning	54	Clarity and strategy sharing (18)
		Synergy between the levels (14)
		Comfort (15)
Physical environment	45	Equipment (13)
		Common areas (7)

**Table IV:** Individual characteristics that influence organizational well-being.

Category	*n*	Specific elements (*n*)
		Being proactive (24)
Being positive	76	Confidence in one's own abilities (17)
		Openness towards the new (14)
		Valuing differences (12)
		Self-motivation and energy (9)
		Openness (23)
		Leadership (19)
Communication	69	Collaborative relationships (16)
		Knowing how to defuse situations (11)
Management of difficulties and conflicts	48	Showing tenacity and refusing to give up (18)
		Striking a balance in tense situations (16)
		Tolerating uncertainty (14)
Socio-emotional skills	29	Creativity (16)
		Empathic communication (13)

### The meaning of well-being in the workplace

The analysis of the responses from 72 subjects identified 248 statements referring to the first issue that flow into four main categories articulated in 14 more specific elements (see Table II).

#### Acceptance of the rules

The first category that stands out is the *acceptance of the rules* (76 statements). In general, it is stated that a clear definition and acceptance of the rules constitutes a fundamental basis of well-being in the workplace. In specific terms, there are four elements.

#### Transparency in communication between colleagues *(26 statements)*

This affirms that when employees openly exchange information in their possession they can work under better conditions both in personal and organizational terms. “Well-being is clear and undistorted communication … sometimes you clash with reality in which the other person instead of streamlining the procedures tends to make them more complicated.”

#### Staying within the boundaries *(20 statements)*

Well-being in the workplace depends on clarity and respect for role boundaries in order to avoid duplications and frictions. In other words, the ability to reduce overlaps and conflicts promotes mutual acceptance and well-being. “Well-being is to be clear about one's role in the organization.”

#### Clarity in the definition of rights and duties by the company *(16 statements)*

Ideally an organization should put in place a system that allows easy recognition of the rights and the duties of each person regardless of their role; it is believed that this could be a source of guidance and both ethical and operational support, steering relationships within the workforce towards a *comfort zone*. “The organization is one entity and should not leave room for ambiguity, there is a certain protocol to be followed. They say that what matters is the result, but things should be done according to certain criteria. There must be strict compliance with protocol, clarity of what you should and you can do, all this leads to worker well-being.”

#### Avoidance of disputes in business relationships *(14 statements)*

It has to be achieved intentionally for the greater good of the company: getting along together, recognizing the unifying aspects rather than those which divide, appreciating differences, are examples of forms of relationships pursued in the name of a common good. “Well-being is remaining calm without clashing with others” or “It is important to find a way to spend the day trying to interact positively with everyone.”

#### Participatory hierarchy

The second category of meaning is represented by *participatory hierarchy* (67 statements), divided into four specific elements.

#### Sharing the company vision *(22 statements)*

It includes a series of statements that highlight the importance of a hierarchy capable of listening and sharing the organizational vision as well its standards. “Well-being is internal communication at all levels from top to bottom in order to understand the objectives is fundamental to working well.”

#### Participatory leadership *(18 statements)*

It comprises a series of statements concerning the organization's ability to foster participatory styles of influence aimed at actively involving employees. “Well-being is the possibility to work with a leader that knows how to inspire people.”

#### Reduction of hierarchical barriers *(15 statements)*

It is the organization's ability to be direct, open, and transparent in the exchange of information. There is also a critical reference to a personalized or paternalistic approach, which contrasts with an expectation of the common definition of goals. “The door should always be open” or “The problem is the presence of two players with equal dignity: we must lower the barriers and well-being comes out.”

#### Leading by example *(12 statements)*

It calls for consistent behaviour, which reinforces the provision for clarity and consistency with common codes in opposition to individualism. “It is not fair to have to put up with arrogant people who act as if they owned the company, each in his own way must lead by example.”

#### Positive relations and working climate

The third category of meaning that emerges consists of *positive relations and working climate* (58 statements). This category contains three specific elements.

#### Knowing how to listen *(23 statements)*

This element refers to a general ability in mutual listening skills and attention to others as a practice that creates positive working conditions. “Well-being is that everyone listen to everyone else.”

#### Being able to rely on the group *(19 statements)*

Constructive relationships can prevail and a positive climate can be created within the working groups. “To experience well-being everyone needs to work in a collaborative group to achieve their goals.”

#### Mental flexibility *(16 statements)*

It describes an arrangement which simplifies problems, by reducing barriers and streamlining interaction which may otherwise be too formal. “Well-being depends on people's mental elasticity. If one is elastic well-being is improved; if one is rigid well-being is adversely affected.”

#### Appreciation of the value of work

The fourth and last category of meaning consists of the *appreciation of the value of work* (47 statements). This category contains three specific elements.

#### Being motivated by work content *(20 statements)*

A part of well-being depends on satisfaction with what employers do. “Well-being is a state of personal growth that occurs when you enjoy what you do.”

#### Economic reward *(16 statements)*

A fair economic reward for the skills and commitment provided fosters the perception of well-being. “If everyone is paid in proportion to what they give you create well-being” or “A just economic reward means feeling valued and recognized.”

#### Job rotation and change in routine role *(11 statements)*

It refers to the ability to vary the experience to avoid the monotony of work. “If you are not lucky enough to have a challenging career the only way to protect well-being is by job rotation.”

### Factors that affect well-being in the workplace

The analysis of the responses from 72 subjects identified 158 statements referring to the second issue, that flow into three main categories articulated into nine more specific elements (see Table III).

#### Values

The first category was defined as *values* (59 statements), which contains three specific elements.

#### Respect *(24 statements)*

It's the acceptance of mutual responsibilities, both professional and personal, as well as acceptance of the value of the organization itself. “Well-being exists where people, organization, principles, and values are respected as a basis for work.”

#### Humility *(19 statements)*

It refers to the definition of a non-judgmental stance towards others and a willingness to communicate regardless of the positions of power, from top to bottom and vice versa. “People are often very proud, they are often lacking in humility … the least competent tend to argue and create problems.”

#### Transparency and exchange of information *(16 statements)*

It is the importance of sharing information in the general interest: if everyone used this method the quality of corporate life would be improved. “An effort must be made to be objective and tolerant in order to understand others, including customers” or “Information should be shared, for example, in meetings how many bosses have basic information.”

#### Organizational functioning

The second category of meaning that emerges is *organizational functioning* (54 statements), which includes three specific elements.

#### Fluid organization *(22 statements)*

It is required in order to define a quality of organizational processes that are linear, without procedural dysfunctions and rigid personal interpretations. “A shared a common goal without too much punctiliousness has a positive effect on the smooth running of the organization.”

#### Clarity and strategy sharing *(18 statements)*

It refers to the organization's ability to place the demands made on individuals within a clear overview and perspective. “There are two levels, managerial and professional: there must be aims and rules which have been agreed at the two levels.”

#### Synergy between the levels *(14 statements)*

It is the organization's general ability to facilitate action over roles. This synergy should result from the motivation of the individual and the organization's desire for achievement. “If I for one understand the dynamics by which the other acts friction is avoided.”

#### Physical environment

The third category of meaning that emerges is defined as the *physical environment* (45 statements), divided into three specific elements.

#### Comfort *(15 statements)*

It's a set of factors (light, heat, space, etc.) that improve the physical quality of permanence in the workplace. “Well-being is also the environment, air, light” or “To have changed location has changed our lives, now we have heat, light, the bathroom.”

#### Equipment *(13 statements)*

It refers to the working instruments which promote both well-being and work activity. “Sometimes details such as the efficiency of computers, the seating position, are important for good health.”

#### Common areas *(seven statements)*

A set of conditions that promote physical well-being through the facilitation of social interaction. “The coffee break with conversation between colleagues is a very important moment” or “The fact that we eat together is positive.”

### Individual characteristics that affect well-being in the workplace

The analysis of the responses from 72 subjects, all in agreement that individual characteristics may influence well-being in the workplace, identified 222 statements referring to the third issue, that flow into four main categories articulated in 14 more specific elements (see Table IV).

#### Being positive

The first category was defined as *being positive* (76 statements). Positivity is an individual attitude that expresses a force arising from self-esteem, confidence and consistency, features that allow a person to contribute to the opening of new horizons, optimism and organizational reliability. In reference to this category five specific elements emerge.

#### Being proactive *(24 statements)*

It refers to a proactive approach towards others and the organization, an active and confident disposition in proposing actions and solutions. “An individual's proactive response tips the balance … provides a positive stimulus.”

#### Confidence in one's own abilities *(17 statements)*

Self-esteem sustained by acquired abilities and by individual competency is described as an attribute that promotes well-being in the workplace. “Feeling able to do the job makes not only me feel good but also others, which leads to respect, meritocracy, rewards from the organization.”

#### Openness towards the new *(14 statements)*

It is a willingness to use knowledge to further new experiences. This element also expressed the employees’ confidence in their ability to improve organizational functioning. “It is always the individual that improves the organization, because otherwise you would become exhausted … There must always be room for novelty.”

#### Valuing differences *(12 statements)*

The appreciation of people with different opinions, or who communicate within different roles is seen as an important aspect of being positive, a variant of the *openness towards the new* which has declined in the context of interpersonal relationships. “Differences are of value within the organization, the inherent rudeness in not respecting the opinions of others is a form of insecurity.”

#### Self-motivation and energy *(nine statements)*

The ability to find one's own stimulus to fuel motivation is seen as a vital factor that reflects positively on well-being. “If a person is radiant and positive, love and passion reflect on well-being” or “An individual characteristic that favours well-being is self-motivation in the sense of caring for the things you do.”

#### Communication

The second category is *communication* (69 statements). The word *communication* is used numerous times and across all dimensions: *good communicator* is a multi-attribute which excites many expectations. In an attempt to focus on a more specific dimension related to the organizational context, four specific elements emerge.

#### Openness *(23 statements)*

It is a dynamic characteristic of sociability and helpfulness in work relationships. “Well-being arises from communication, being able to communicate, sitting around a table with colleagues is important because the discussions often lead to a solution.”

#### Leadership *(19 statements)*

Subjects use this word to describe the ability to influence and lead the group in a positive manner, and also to define authoritative behaviour. “If each in his own small way exercised leadership it would benefit everyone” or “Being open but self-confident, not backing down in front of obstacles means not immobilizing the organization.”

#### Collaborative relationships *(16 statements)*

It refers to the disposition towards listening to and understanding others in the search for common solutions. “It's important to find a way to spend the hours of the day trying to interact well, the way we relate to others affects everyone … We should be ready to help, be polite.”

#### Knowing how to defuse situations *(11 statements)*

It is the tendency, even jokingly and/or self-deprecatingly, to diminish problems without denying their existence. “An important attribute is an individual's capacity for fearless self-criticism” or “A joke reduces conflict and barriers! I would also like to think with joy of work.”

#### Management of difficulties and conflicts

The third category of meaning that emerges refers to the *management of difficulties and conflicts* (48 statements). Stamina and the ability to manage on the occasions when requirements appear to exceed resources creating the inevitable tensions are described as attributes that help an individual go through organizational life generating positivity and protecting basic well-being. Three specific elements emerge.

#### Showing tenacity and refusing to give up *(18 statements)*

To be determined and not to become discouraged are described as individual characteristics that ensure the rewards of well-being. “There are times when if you give up your objectives, you will collapse, it is better to be reactive.”

#### Striking a balance in tense situations *(16 statements)*

The ability to mediate is described as being positive both for the employees and the company, it means being an active participant without being drawn into conflicts or tensions. “We must be able to clear-up group tensions and misunderstandings. We must never lose hope in the power of communication.”

#### Tolerating uncertainty *(14 statements)*

This element is described as the ability to maintain a positive response to the working environment even in conditions of relative discomfort and accept uncertainty whilst awaiting new opportunities. “Well-being means being able to stay calm, not being under pressure and offering the same thing to others.”

#### Socio-emotional skills

The fourth category of meaning that emerges is that of *socio-emotional skills* (29 statements). This is an area that refers to features perceived as specials: these features have in common a facilitating role of individual and group functions. Within this category two specific elements can be found.

#### Creativity *(16 statements)*

It refers to the use of imagination in dealing with problems and the ability to see problems from new angles. “An individual that has creativity can assist the organization, can open doors and consequently facilitate the disclosuring of the other as persons.”

#### Empathic communication *(13 statements)*

It is described as the subjects’ ability to express a closeness that catches the sense and rearranges emotionally the dynamic field of the individual-group-organization interaction. “We need to identify with the person in front of us to understand their needs. Well-being is a closeness between me and the other person.”

### Additional considerations that emerged from individual interviews

The nine individual interviews, as well as providing material for the three research questions discussed so far, allowed for the introduction of additional analysis that called into question the dynamics of the concept of well-being. In particular, the analysis of the sample episodes narrated by the subjects allowed us to focus on two aspects.

The first aspect is referred to the conviction that well-being in the workplace is *a phenomenon in which two directions of organizational operation are dynamically integrated*. The first direction—using a participant's words—is the “Organization as a structure that exists regardless of each individual”, that is, a top-down direction formed by structures, decisions, and a work ethic built-up over time. The second direction is formed by the organizational actions of individuals and groups, or a bottom-up direction that could—if embraced by many individuals—become long-term well-being. “If it's true that organization is something that you receive from others is also true that it is something you can offer to others: this promotes general well-being” or “You construct well-being yourself, but if you have worked successfully those who come after you will recognize it.”

The second aspect is referred to the conviction that niches of prosperity can be created individually through people who make up the working groups, for example, a participant said he was rather bewildered when he first arrived at the company and had only begun to “Breathe an air of good management” when he actually met, in the section in which he worked, people with whom he could collaborate constructively.

Furthermore, it is evident that if some people tend to represent well-being in a dichotomous manner, separating an ideal from a real dimension, so that judgment may tend to focus on one of these two polarities forgetting the other, some others are able to overcome this simplification asserting that the well-being is dynamically placed between the level of *what should be* and the level of *what is*. “One cannot truly understand well-being without an overview because it always works on two levels, that of desires and that of possibilities.”

## Discussion

The results obtained from the study are consistent with the indications in literature about both the interaction between individual and organization in the construction of well-being in the workplace and indication of some individual characteristics as cooperating in the formation of well-being (Graham & Shier, 2011; Hodkinson et al., 2004). Data are also consistent with the recognized importance of social constructivism in research concerning the individual in the corporate context (Loftus & Higgs, 2010) and also confirm Allcorn's hypothesis (1995) concerning the use of subjective points of view for understanding well-being in the workplace and the projected change of organizational culture in a direction favourable to the creation of well-being.

More specifically, the research was divided into three parts. The answers to the first research question, the meaning of well-being in the workplace, showed a perception of well-being characterized as interaction between people and organizations, in accordance with the claims made by some authors (Alvesson & Willmott, 2002; Mininni et al., 2010).

The first category highlighted—*acceptance of the rules*—clearly expresses a perception that corporate rules are described as a source of well-being if there is interplay of transparency and organizational behaviour. The second category—*participatory hierarchy*—emphasizes that the lack of barriers, sharing and involvement in hierarchical relationships is seen as an element of well-being. The third category—*positive relations and working climate*—returns once again to the perception of well-being as a positive quality of interpersonal relationships, regardless of the rules and position held. The fourth category—*appreciation of the value of work*—shows that structural factors are linked to employee satisfaction towards the content of their job, towards their salaries, and towards social and cognitive stimulation. In this category we find a relation between job satisfaction and well-being as described by many authors (Harris et al., 2003; Ter Doest et al., 2006). Overall, we can see that well-being is perceived not only as an interaction between individuals and the organization, but is strongly related to the quality of the relationship between individuals. This is in agreement with what is said by Settoon and Mossholder (2002), who described how the quality of relationships in the working environment is predictive of results-oriented OCBs and respect for the individual, and by D'Amato and Zijlstra (2008), in a study conducted in Europe on the staff of 406 hospitals which revealed how individual characteristics linked to the working environment and to self-efficacy were a prerequisite for satisfactory results and a consequent increase in well-being.

The second research question, factors that influence well-being in the workplace, was deliberately placed in general terms, so as to allow participants the freedom to decline the definition of the factors influencing well-being in objective or subjective, individual, organizational, or structural terms.

The first category that emerged—*values*—can be seen as a set of ethical and communicational features that the individual and the group would have to be encouraged to express in order to benefit overall well-being. This seems to confirm existing positions on the importance of individual values on job satisfaction and on the working environment (Bulger, Matthews, & Hoffman, 2007; Burke, 2000; Diskienė & Goštautas, 2010). The second and the third categories—*organizational functioning* and *physical environment*—refer to more objective elements of the organization, although some specific elements such as *clarity and common strategies* and *common spaces* refer instead to aspects of communication.

The third research question, individual characteristics that affect well-being in the workplace, has confirmed several previous studies concerning individual factors considered to be favourable to well-being.

In particular, in the category *being positive*, the perception that an active and trusting attitude in proposing solutions and positive action is a feature which affects well-being is in line with the results of studies that describe the link between positive emotions, the ability to achieve satisfaction at work, and the ability to be open to organizational change (De Neve & Copper, 1998; Fineman, 2006; Lent, 2004; Linley et al., 2010). The specific elements that arose regarding confidence in the respondents’ own resources and self-motivation are consistent with the literature that examines the issue of self-esteem as a resource for well-being of both the individual and the organization (Feldt et al., 2006; Kalimo et al., 2002; O'Brien-Wood, 2001; Pierce & Gardner, 2004; Pulkkinen et al., 2006).

Similarly, in the second category *communication*, the perception of respondents agrees with recent literature concerning interaction as an area linked to relational well-being. The perception that openness in relationships and collaborative relationships are factors influencing well-being in the workplace is in line with that stated by Butler and Waldroop (2004), Kamdar and Van Dyne (2007), and Kumar et al. (2009) about extroversion and pleasantness in interpersonal relationships as factors supporting OCBs. In turn, the perception that leadership is influential with regard to well-being agrees with research conducted by Purkiss et al. (2008). Finally, the ability to defuse a situation is consistent with that described by George and Jones (1997) concerning the positive effect of certain extra role behaviours in the workplace.

In the third category *management of difficulties and conflicts*, resilience or the ability to cope with conflicting tensions or difficulties of different kinds is described as a characteristic that aids a person during their working life, generating positivity or at least protecting their basic well-being. This perception agrees with the importance of political skills in adapting positively to the workplace (Ferris et al., 2000; Perrewé et al., 2000). The quality of perseverance, as well as the ability to remain well-balanced in a dispute are considered both within the context of political skills and as a personality traits favouring OCBs (Borman & Penner, 2001). In addition, all three elements which emerged in this category confirmed the most recent views on resilience as a promoter of well-being in the workplace (Cooper, 2010; Ferguson, 2009; Magrin, 2008).

The fourth category of *socio-emotional skills* is instead connected with characteristics perceived as specials. This peculiarity seems to stem from a facilitating function performed by these capacities in respect of the working environment, as is referred to, for example, in a definition (“Knowing how to ‘wash away’ the problems within the organization”) contained in a statement relative to this item of empathic communication. This category appear to find confirmation in literature only in an indirect sense, such as occurs with the empathy necessary to improve the relationship with a patient (and consequently between the members of staff) in health care services (Hojat, 2009).

Finally, the considerations which emerged concerning the *niches of well-being* in the individual interviews are consistent with the observations of Van De Vliert (2008) when he says that “at the lowest levels, each employee adapts his or her own well-being to a mosaic of working conditions, group characteristics, and organizational circumstances, which is perhaps hardly shaped climate and wealth” (p. 524).

## Conclusions

The research results provide feedback as to how each variable is perceived by the individual as a useful resource for improving well-being in the workplace. These findings could define people as *activators* of well-being and can be placed in supplementary terms compared to the vision of people considered as *receptors* of well-being from the external environment. In that sense, well-being in the workplace could be promoted not only *from above* through objective action by management, for example, the promotion of organizational welfare policies, but also *from below*, through the transformation of individual traits and behaviours that are manifested in people's activities (Graham & Shier, 2010; Hodkinson et al., 2004; Loftus & Higgs, 2010).

At an applied level, the data obtained through the research confirms the possibility of active involvement by people in the construction of well-being, within this organizational vision that is capable of integrating bottom-up and top-down processes. More precisely, it is possible to identify three areas of intervention.

The first area is represented by the continuous training about well-being in the workplace as a result of constructive collaboration between the individual, group, and organization. This issue could also enhance a series of bottom-up initiatives focused on organizational climate, avoiding in this respect initiatives in which the individual is perceived as a mere passive recipient, but rather by exploiting the approach of action research.

A second area may consist of organizational development initiatives aimed at small groups, leaders and other strategic players in the construction of the subsystems of well-being in the workplace. Furthermore, workplace counselling initiatives can be contemplated, aimed at reducing stress and improving proactive adaptation to the workplace.

Finally, a third area of action consists of research aimed at investigating perceptions of well-being among the different roles in order to provide useful monitoring to the human resources management team and instigate organizational change. In this case also a qualitative approach, capable of encouraging participation through projects of investigation that use interviews and focus groups, can be regarded as more consistent with the results of the study presented here.

It should be remembered that the research was limited by the participants involved and by the sole use of the paper and pencil technique of data analysis. In this sense, future studies could be undertaken in other organizational contexts with the object of enriching the data base available to scholars and facilitate the identification of further individual characteristics which contribute to well-being in the workplace. Furthermore, a more ample data base could facilitate the use of software for the analysis of the content (e.g., ALCESTE) and enabling the comparisons between the perceptions of groups composed of participants where distinction is made according to their organization of belonging, their role, and other socio-demographic characteristics.

## References

[CIT0001] Allcorn S (1995). Understanding organizational culture as the quality of workplace subjectivity. Human Relations.

[CIT0002] Alvesson M, Willmott H (2002). Identity regulation as organizational control: Producing the appropriate individual. Journal of Management Studies.

[CIT0003] Bambacas M, Patrickson M (2008). Interpersonal communication skills that enhance organisational commitment. Journal of Communication Management.

[CIT0004] Bandura A (1986). Social foundation of thought and action: A social cognitive theory.

[CIT0005] Borman W. C, Penner L. A, Roberts B. W, Hogan R (2001). Citizenship performance: Its nature, antecedents, and motives. Personality psychology in the workplace. Decade of behaviour.

[CIT0006] Brunstein J. C, Schultheiss O. C, Grässmann R (1998). Personal goals and emotional well-being: The moderating role of motive dispositions. Journal of Personality and Social Psychology.

[CIT0007] Bulger C. A, Matthews R. A, Hoffman M. E (2007). Work and personal life boundary management: Boundary strength, work/personal life balance, and the segmentation-integration continuum. Journal of Occupational Health Psychology.

[CIT0008] Burke R (1993). Organizational-level interventions to reduce occupational stressors. Work & Stress: An International Journal of Work.

[CIT0009] Burke R (2000). Do managerial men benefit from organizational values supporting work-personal life balance?. Women in Management Review.

[CIT0010] Butler T, Waldroop J (2004). Understanding ‘people’ people. Harvard Business Review.

[CIT0011] Cable D. M, Judge T. A (1996). Person-organization fit, job choice decisions, and organizational entry. Organizational Behavior and Human Decision Processes.

[CIT0012] Cooper C. L (2010). The happiness agenda. Stress and Health.

[CIT0013] Csikszentmihályi M (1997). Finding flow: The psychology of engagement with everyday life.

[CIT0014] Custers R, Aarts H (2005). Positive affect as implicit motivator: On the nonconscious operation of behavioral goals. Journal of Personality and Social Psychology.

[CIT0015] D'Amato A, Zijlstra R. H (2008). Psychological climate and individual factors as antecedents of work outcomes. European Journal of Work and Organizational Psychology.

[CIT0016] De Neve K. M, Copper H. H (1998). The happy personality: A meta-analysis of 137 personality traits and subjective well-being. Psychological Bulletin.

[CIT0017] Di Nuovo S, Zanchi S (2008). Work well-being: A study on satisfaction and positive emotions in job. Giornale di Psicologia.

[CIT0018] Diskienė D, Goštautas V (2010). Relationship between individual and organizational values and employees. Job satisfaction Business.

[CIT0019] Feldt T, Mäkikangas A, Aunola K, Pulkkinen L, Kaprio J, Rose R (2006). Sense of coherence and optimism: A more positive approach to health. Socioemotional development and health from adolescence to adulthood.

[CIT0020] Ferguson D (2009). Resilience: What you need to know about personal wellbeing. Teacher.

[CIT0021] Ferris G. R, Perrewe P. L, Anthony W. P, Gilmore D. C (2000). Political skill at work. Organizational Dynamics.

[CIT0022] Fineman S (2006). On being positive: Concerns and counterpoints. The Academy of Management Review.

[CIT0023] Fligstein N (1997). Social skill and institutional theory. American Behavioral Scientist.

[CIT0024] French J. R. P, Caplan R. D, Van Harrison R (1982). The mechanism of job stress and strain.

[CIT0025] Gabriel Y (2000). Storytelling in organizations: Facts, fictions, and fantasies.

[CIT0026] George J. M, Jones G. R (1997). Organizational spontaneity in context. Human Performance.

[CIT0027] Graham J. R, Shier M. L (2010). Social work practitioners and subjective well-being: Personal factors that contribute to high levels of subjective well-being. International Social Work.

[CIT0028] Graham J. R, Shier M. L (2011). Mindfulness, subjective well-being, and social work: Insight into their interconnection from social work practitioners. Social Work Education.

[CIT0029] Grant S. L, Langan-Fox J, Anglim J (2009). The big five traits as predictors of subjective and psychological well-being. Psychological Reports.

[CIT0030] Gross J. J (1998). The emerging field of emotion regulation: An integrative review. Review of General Psychology.

[CIT0031] Gross J. J (2006). Handbook of emotion regulation.

[CIT0032] Guest D (2002). Human resource management, corporate performance and employee wellbeing: Building the worker into HRM. Journal of Industrial Relations.

[CIT0033] Guest D, Conway N, Schabracq M, Winnubst J, Cooper C (2002). The psychological contract at work and its effects on health and well-being. Handbook of Work and Health Psychology.

[CIT0034] Harris C, Daniels K, Briner R. B (2003). A daily diary study of goals and affective well-being at work. Journal of Occupational and Organizational Psychology.

[CIT0035] Harter J. K, Schmidt F. L, Keyes C, Keyes C, Haidt J, Seligman M (2003). Well-being in the workplace and its relationship to business outcomes: A review of the Gallup studies. Flourishing: Positive psychology and the life well-lived.

[CIT0036] Haslam H, Whelan J, Bastian B (2009). Big Five traits mediate associations between values and subjective well-being. Personality and Individual Differences.

[CIT0037] Hochwarter W. A, Thompson K. R (2010). The moderating role of optimism on politics-outcomes relationships: A test of competing perspectives. Human Relations.

[CIT0038] Hodkinson P, Hodkinson H, Evans K, Kersh N, Fuller A, Unwin L (2004). The significance of individual biography in workplace learning. Studies in the Education of Adults.

[CIT0039] Hojat M (2009). Ten approaches for enhancing empathy in health and human services cultures. Journal of Health and Human Services Administration.

[CIT0040] Hughes J (2005). Bringing emotion to work emotional intelligence, employee resistance and the reinvention of character. Work Employment & Society.

[CIT0041] Isen A. M, Berkowitz L (1987). Positive affect, cognitive processes, and social behaviour. Advances in experimental social psychology.

[CIT0042] Judge T. A, Heller D, Klinger R (2008). The dispositional sources of job satisfaction: A comparative test. Applied Psychology: An International Review.

[CIT0043] Kalimo R, Pahkin K, Mutanen P (2002). Work and personal resources as long-term predictors of well-being. Stress and Health.

[CIT0044] Kamdar D, Van Dyne L (2007). The joint effects of personality and workplace social exchange relationships in predicting task performance and citizenship performance. Journal of Applied Psychology.

[CIT0045] Krueger R. A, Casey M. A (2000). Focus groups.

[CIT0046] Kumar K, Bakhshi A, Rani E (2009). Linking the big five personality domains to organizational citizenship behavior. International Journal of Psychological Studies.

[CIT0047] Kyngas H, Vanhanen L (1999). Content analysis as a Research Method. Hoitotiede.

[CIT0048] Lawson K. J, Noblet A. N, Rodwell J. J (2009). Promoting employee wellbeing: The relevance of work characteristics and organizational justice. Health Promotion International.

[CIT0049] Lent R. W (2004). Toward a unifying theoretical and practical perspective on wellbeing and psychosocial adjustment. Journal of Counseling Psychology.

[CIT0050] Lent R. W, Brown S. D, Lent R. W (2008). Understanding and promoting work satisfaction. An integrative view. Handbook of counseling psychology.

[CIT0051] Lent R. W, Brown S. D (2006). Integrating person and situation perspectives on work satisfaction: A social-cognitive view. Journal of Vocational Behavior.

[CIT0052] Lent R. W, Fouad N. A, Hartung P. J, Subich L. M (2011). The self as agent in social cognitive career theory. Developing self in work and career.

[CIT0053] Linley P. A, Harrington S, Garcea N (2010). Oxford handbook of positive psychology and work.

[CIT0054] Loftus S, Higgs H (2010). Researching the individual in workplace research. Journal of Education and Work.

[CIT0055] Magrin M. E (2008). From resistance to resilience: Promoting wellbeing in the workplace. Giornale Italiano di Medicina del Lavoro ed Ergonomia.

[CIT0056] Mininni G, Manuti A, Scardigno R, Rubino R (2010). Subjective wellbeing between organizational bonds and cultural contaminations. World Futures: The Journal of Global Education.

[CIT0057] Murray M (2002). Connecting narrative and social representation theory in health research social sciences. Social Science Information.

[CIT0058] O'Brien-Wood C. P (2001). Coping with organizational change: Adaptiveness versus trait and change-related coping in predicting employee well-being *(Doctoral dissertation)*.

[CIT0059] Perrewé P. L, Ferris G. R, Funk D. D, Anthony W. P (2000). Political skill: An antidote for workplace stressors. Academy of Management Executive.

[CIT0060] Pierce J. L, Gardner D. G (2004). Self-esteem within the work and organizational context: A review of the organization-based self-esteem literature. Journal of Management.

[CIT0061] Pulkkinen L, Feldt T, Kokko K (2006). Adaptive behavior in childhood as an antecedent of psychological functioning in early middle age: Linkage via career orientation. Social Indicators Research.

[CIT0062] Purkiss R. B, Rossi R. J, Glendon A. I, Thompson B. M, Myors B, Glendon A, Thompson B. M, Myors B (2008). Sense of community: A vital link between leadership and wellbeing in the workplace. Advances in organisational psychology.

[CIT0063] Ryan R. M, Deci E. D (2000). Self-determination theory and the facilitation of intrinsic motivation, social development, and well-being. American Psychologist.

[CIT0064] Settoon R. P, Mossholder K. W (2002). Relationship quality and relationship context as antecedents of person- and task-focused interpersonal citizenship behavior. Journal of Applied Psychology.

[CIT0065] Sheu H.-B, Lent R. W (2009). A social cognitive perspective on well-being in educational and work settings: Cross-cultural considerations. International Journal for Educational and Vocational Guidance.

[CIT0066] Ter Doest L, Maes S, Gebhardt W. A, Koelewijn H (2006). Personal goal facilitation through work: Relationships with employee satisfaction and wellbeing. Applied Psychology: An International Review.

[CIT0067] Terrion J. L, Ashforth B. E (2002). From ‘I’ to ‘we’: The role of putdown humor and identity in the development of a temporary group. Human Relations.

[CIT0068] Van De Vliert E, Cartwright S, Cooper C. L (2008). Climato-economic niches of employee well-being. The Oxford handbook of organizational well being.

[CIT0069] Warr P, Kahneman D, Deiner E, Schwarz N (1999). Well-being and the work place. Well-being: The foundations of hedonic psychology.

[CIT0070] Wright T. A, Cropanzano R, Bonett D. G (2007). The moderating role of employee positive well being on the relation between job satisfaction and job performance. Journal of Occupational Health Psychology.

[CIT0071] Zapf D (2002). Emotion work and psychological well-being: A review of the literature and some conceptual considerations. Human Resource Management Review.

